# Adults' Perceived Prevalence of Enteric Fever Predicts Laboratory-validated Incidence of Typhoid Fever in Children

**Published:** 2007-12

**Authors:** Xinguang Chen, Bonita Stanton, Al Pach, Andrew Nyamete, R. Leon Ochiai, Linda Kaljee, Baiqing Dong, Dipika Sur, S.K. Bhattacharya, Siti Sapardiyah Santoso, Magdarina Agtini, Zahid Memon, Zulfiqar Bhutta, Canh Gia Do, Lorenz von Seidlein, John Clemens

**Affiliations:** 1 Carman and Ann Adams Department of Pediatrics, Wayne State University School of Medicine, Detroit, MI 48201, USA; 2 International Vaccine Institute, Seoul, Republic of Korea; 3 Department of Pediatrics, University of Maryland School of Medicine, Maryland, USA; 4 Guangxi Centers for Disease Control and Prevention, Guangxi, China; 5 National Institute of Cholera & Enteric Diseases, Kolkata, India; 6 National Institute of Health Research and Development, Jakarta, Indonesia; 7 Department of Pediatrics, Aga Khan University, Karachi, Pakistan; 8 National Institute of Hygiene and Epidemiology, Nanoi, Viet Nam

**Keywords:** Developing countries, Incidence, Perceptions, Prediction model, Prevalence, Salmonella, Typhoid fever, China, India, Viet Nam, Indonesia, Pakistan

## Abstract

This study was undertaken to develop a model to predict the incidence of typhoid in children based on adults’ perception of prevalence of enteric fever in the wider community. Typhoid cases among children, aged 5-15 years, from epidemic regions in five Asian countries were confirmed with a positive *Salmonella* Typhi culture of the blood sample. Estimates of the prevalence of enteric fever were obtained from random samples of adults in the same study sites. Regression models were used for establishing the prediction equation. The percentages of enteric fever reported by adults and cases of typhoid incidence per 100,000, detected through blood culture were 4.7 and 24.18 for Viet Nam, 3.8 and 29.20 for China, 26.3 and 180.33 for Indonesia, 66.0 and 454.15 for India, and 52.7 and 407.18 for Pakistan respectively. An established prediction equation was: incidence of typhoid (1/100,000= −2.6946 + 7.2296 × reported prevalence of enteric fever (%) (F=31.7, p<0.01; R^2^=0.992). Using adults’ perception of prevalence of disease as the basis for estimating its incidence in children provides a cost-effective behavioural epidemiologic method to facilitate prevention and control of the disease.

## INTRODUCTION

Typhoid fever remains a significant threat to the health of individuals in developing countries. Although its prevalence varies across regions (1–5), the disease causes an estimated 21.6 million illnesses and 216,500 deaths globally each year (1). Effective control of typhoid fever in developing countries represents a significant public-health challenge (6–10). Epidemiological data, especially data on the incidence of typhoid, are needed for making effective decisions to plan prevention programmes and for the evaluation of programme effects (6,11). Incidence of a disease can be used for assessing the number of new cases occurring in a given time period and for predicting the number of new cases in the years to come, if no additional disease-control measures are taken. An unacceptably high rate and/or an increase in the incidence of an infectious disease suggest the need for a greater effort to prevent the disease, such as a vaccine campaign. Data on incidence are also needed for vaccine producers to plan production of their vaccines. After the implementation of a prevention campaign, decline in the incidence of the disease is typically the outcome sought as evidence supporting the effectiveness of the intervention.

The importance of incidence data for the prevention and control of infectious diseases has long been recognized (10,11). However, data on the incidence of typhoid are rare in most developing countries. Measurement of infectious diseases in developing countries is often based on clinical symptoms rather than laboratory confirmation of pathogenic agents (10,12–16). Measuring the incidence of an illness, such as typhoid fever for which clinical symptoms are not specific, is a significant challenge to control and prevention of infectious diseases in developing countries (12,17). Valid measurement of incidence of typhoid requires a sustained and laboratory-based disease-surveillance system with extensively-trained personnel, adequate resources, and gold standard for diagnosis (11). Despite much progress in recent years, developing countries remain subject to limitations in infrastructure, financial resources, and trained personnel (13,15,17,18). Accurate detection of infection due to S. Typhi requires complex procedures of both blood or bone-marrow cultures with blood and bone-marrow samples collected at different periods (19,20). Avoidance of false positive among paediatric patients is an additional concern (21).

While conducting studies for the Diseases of the Most Impoverished (DOMI) Programme in several Asian countries (8), we collected data on the incidence of typhoid among children aged 5-15 years based on its laboratory confirmation. In a separate but related sociobehavioural study, we randomly collected sampled household-survey data on perceived prevalence of enteric fever. The purpose of this analysis was to establish a method that can predict the incidence of typhoid fever in children using perceived prevalence data collected from adults. We hypothesized that perceptions of the frequency of a significant disease, such as typhoid fever, in children by adults who live in the same community would be closely related to the actual frequency of its occurrence in children.

## MATERIALS AND METHODS

### Study sites and population

**Fig. 1:**
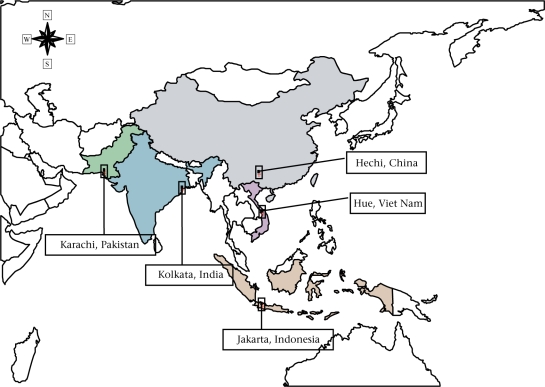
Geographic location of the five study sites in China, India, Indonesia, Pakistan, and Viet Nam

Data for the study were collected in five Asian countries—China, India, Pakistan, Indonesia, and Viet Nam. One county-level administrative area was selected from each participating country to be included in the study: Hechi, China; Kolkata, India; North Jakarta, Indonesia; Karachi, Pakistan; and Hue, Viet Nam. (a) Hechi is a county of Guangxi Zhuang Autonomous Region (or Guangxi province) located in southwest China. The site includes an urban township (Jin-Cheng Jiang) and a rural township (Dong Jiang). (b) The study site in India consists of Ward 29 and Ward 30 in Kolkata (formally Calcutta) in West Bengal. Kolkata is the third largest city in India. The two neighbouring wards included cover several *bustees*, characterized by legally-recognized and registered urban slums. (c) The study site in Pakistan includes three squatter settlements (Hijrat Colony, Sultanabad, and Bilal Colony) in Karachi. The Hijrat Colony and the Sultanabad are located in the central urban area and the Bilal Colony is located in the industrial area of Karachi. (d) The study site in Indonesia includes the urban slums of Tanjung Priok and Koja in North Jakarta. (e) The study site Hue City is located in central Viet Nam. Hue covers both urban and semi-urban areas and is divided by the Perfume River that runs into the East China Sea. The geographic locations of the five study sites are marked in Figure 1, and the general characteristics of these sites, including total population and per-capita gross national income (GNI), are summarized in Table 1.

Although the five study sites are located in different regions in Asia and differ from each other in many respects, including level of economic development GNI, age composition of the population, and educational attainment, typhoid fever is prevalent in all the five sites, and there is a lack of a systematic prevention-intervention effort specifically targeting the disease. For the purpose of this study, per-capita GNI and educational attainment of the population were included in the modelling analysis. Other factors, e.g. sanitary conditions, lifestyles, and overcrowding, associated with enteric fever were not included because measures across the five study sites were not available.

### Sampling

Samples for the sociobehavioural household surveys were randomly selected from all the study sites. Within the five study sites, the rural villages and urban and semi-urban residential areas where typhoid fever was common were chosen for sampling. For study sites where disease statistics was available (China and India), residential areas with high rates of enteric fever were selected; for other sites where such data were lacking (Indonesia and Pakistan), local health administrators and primary health workers were contacted to determine residential areas with high rates of enteric fever; and for the site in Viet Nam, 13 urban and semi-urban communes were randomly selected from the 24 communes in Hue, a typhoid fever epidemic region. Households were randomly selected to participate in the survey using population census data, and the household head or his/her spouse was then randomly selected from the sampling frame for interviewing. This method of randomizing a particular respondent within a household was used for ensuring a more equal number of male and female participants. The participation rates were 95% for Hechi, China, 94% for Kolkata, India, 100% for North Jakarta, Indonesia, 100% for Karachi, Pakistan, and 89% for Hue, Viet Nam.

### Data on incidence of typhoid fever

Incidence of typhoid was the dependent variable for the study. Data used for computing the incidence rate was based on laboratory-validated typhoid cases and census population data. New typhoid cases were derived from the disease-surveillance systems of the selected study sites, which were supported by the DOMI Programme. New cases were identified from all febrile patients who had fever for three or more days. These patients were captured either when they visited health service facilities (clinic or health station as in China, Indonesia, and Viet Nam) where healthcare was easily feasible and accessible or when they were referred to the related health service providers by community health workers who conduct household visits either on a weekly (Pakistan) or on a monthly basis (India) where it is hard for patients to visit healthcare facilities.

Ten mL of blood was collected from all the captured febrile patients for laboratory culture to detect S. Typhi as the evidence supporting the diagnosis of typhoid fever. The blood sample was inoculated in sterile Bactec Plus¯ or Pediatric Bactec Plus¯ (Becton Dickenson, USA) bottles and processed to identify S. Typhi. When bacterial growth was confirmed, the blood culture bottle was subcultured on MacConkey agar plate, irrespective of Gram stain. Even with no visible growth, the bottle was subcultured on MacConkey agar on day 1, 2, 4, and 7. The bottles were incubated for up to 10 days before discarding. Additionally, all presumptive S. Typhi isolates were sent to the reference laboratory (Wellcome Trust Research Unit, Ho Chi Minh City, Viet Nam) for confirmation of culture. No discrepancies were found in the results of blood culture between the reference laboratory and the site laboratories. Consequently, a febrile case with blood culture positive for S. Typhi was diagnosed as a case of typhoid fever. Data used for this analysis included all new cases detected in a one-year period. Incidence rate (1/100,000) was computed as the ratio of total new cases of five through 15 years old in one year over the total population in the same age range. We computed the incidence for the age range of 5-15 years because data only for this age range were collected across the five study sites.

### Data on perception of disease

A standardized questionnaire was used for collecting data on perception of enteric fever through face-to-face interviews (22). The questionnaire was developed in English with information from the qualitative study. It was subsequently translated into the languages spoken by the people in each of the five study sites and culturally and ethnically adjusted for use there. Back translation was used for ensuring the accuracy of the translation. Local terms for enteric fever were identified through the qualitative pilot studies and were used in the interview. Four questions assessing perceptions of the disease common across the five study sites were: (a) “How common do you think enteric fever is in your village/town? (1=never occurred, 2=not very common, 3=common, and 4=very common; in Viet Nam, a response of ‘not common’ was used in the places of responses 1 and 2)”. Responses to this question were dichotomized (1 and 2 recoded as negative, 3 and 4 recoded as positive) and used for assessing the perceived prevalence of enteric fever in the wider community. (b) “Has anyone in your household suffered from enteric fever (1=yes, and 2=no)?” Responses to this question were used for assessing the prevalence of disease based on experience of disease among household members. (c) “Have you known anyone personally (including relatives, friends, and acquaintances, other than your household members) who has been sick due to enteric fever? (1=yes, 2=no)” Responses to this question were used for assessing the perceived prevalence based on knowledge of experience of disease among people other than family members. (d) “Have you known anyone personally (other than your household members) who was died of enteric fever? (1=yes, 2=no)”. Responses to this question were used for assessing the prevalence of disease based on knowledge of death due to enteric fever. Percentage was computed for these four variables and used for building the prediction model; the first variable was selected as the predictor variable for modelling because of its close correlation with incidence rate—the dependent variable.

### Ethics

Written informed consent was obtained from all respondents, following the protocols approved by agencies in the participating countries in charge of the protection of human subjects in research and by the Secretariat Committee for Research Involving Human Subjects, World Health Organization, Geneva, Switzerland. Participation in the study was voluntary, and confidentiality was ensured at all times.

### Statistical analysis

Since the purpose of this analysis was to establish a model that would correlate, with a high degree of closeness, the perceived prevalence data by adults with the actual incidence data based on the laboraoty-verified typhoid fever, we used the following basic prediction model:

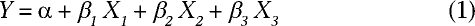

Where *Y*=incidence of typhoid based on laboratory-validated disease data and census population data; *X*_1_=perceived prevalence of enteric fever in the wider community (if enteric fever was thought to be common or very common in the village/town) based on survey data; *X*_2_ and *X*_3,_=other predictor variables; and β_1_, β_2_, and β_3_ were regression coefficients.

In searching a best prediction model, we first analyzed the four variables that assessed the perceived prevalence of the disease by plotting the rates of prevalence of these measures with the incidence. Guided by findings from this bivariate analysis, one prevalence-perception variable, e.g. whether enteric fever was common in the wider community, was used in establishing the prediction model. We included two additional predictor variables (*X*_2_=per-capita GNI and *X*_3_=educational attainment of the population) to take into account cross-country differences in socioeconomic status, because these two factors may affect perception of disease for people who live in different socioeconomic settings. To search for the reliable, valid, and simplest prediction equations, we tested the proposed model (1) above in two steps: we first tested the model with only *X*_1_ being included; we then tested the models with the addition of other possible predictor variables one at a time, including gross per-capita GNI (in US$ 100) and educational attainment of the population (percentage of people with less than high school education).

In addition to using the incidence rate as *Y*, a logit transformation of the incidence was used in the modelling analysis. With the logit transformation of *Y*, model (1) was converted into a logit (or log linear) model, which is often used in prediction analysis of rates (23):

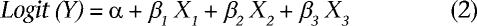



In both models (1) and (2), the incidence *Y* and *logit(Y)* were used as the dependent variable. In conducting the modelling analyses, the F-test was used for assessing the reliability of a regression model when the model parameters were estimated with data, the Student's t-test was used for assessing the significance of regression coefficients associated with the predictor variables, and the statistic R^2^ (percentage of the variance of the dependent variable explained by the model) was used for assessing the goodness-of-fit of a model to the data (24). The prediction equation with the largest *R*^2^ value was used as the criteria for the selection of a ‘best’ model for practical use.

## RESULTS

### Characteristics of the survey study samples

Data from 3,300 subjects, aged 18-60 years, from the five study sites were included in this analysis: 624 from China; 561 from India; 591 from Indonesia; 481 from Pakistan; and 1,043 from Viet Nam. Their mean age was 38.7 (SD=7.6) for China, 36.7 (SD=11.2) for India, 37.3 (SD=7.5) for Indonesia, 33.2 (SD=9.7) for Pakistan, and 40.2 (SD=9.7) years for Viet Nam. Detailed data on the total population, and age and gender compositions, marital status, and the per-capita GNI by study sites are presented in Table 1.

**Table 1:** Characteristics of study sites, study population, and sample for survey study

	Study sites				
Characteristics	Hechi, China	Kolkata, India	N. Jakarta, Indonesia	Karachi, Pakistan	Hue, Viet Nam

Population	98,103	56,946	160,261	41,845	84,488
Per-capita GNI (US$)	1,100	540	810	520	480
Sample size (N)	624	561	591	481	1,043
Gender (%)					
Male	48.9	51.9	49.9	47.4	39.0
Female	51.1	48.1	50.1	52.6	61.0
Age (years) (%)					
<40	58.6	59.5	66.8	71.3	48.8
40+	41.4	40.5	33.2	28.7	51.2
Mean (SD)	38.7 (7.6)	36.7 (11.2)	37.3 (7.5)	33.2 (9.7)	40.2 (7.6)
Education (%)					
Less than high school	80.8	81.0	47.7	50	56.5
High school and plus	19.2	19.0	52.3	50	43.5
Marital status (%)					
In marriage of all participants	99.0	78.8	97.3	84.2	94.9

GNI=Gross national income; SD=Standard deviation

### Validated incidence of typhoid and perceived prevalence of enteric fever

In total, 21,380 febrile episodes were documented among 159,951 children, aged 5-15 years, from the five study sites. Of these febrile cases, 266 typhoid cases were diagnosed with an average incidence of 157.11/100,000. The incidence rate varied substantially across the study sites (Table 2) with 24.18/100,000 for Viet Nam, 29.20/100,000 for China, 180.33/100,000 for Indonesia, 407.18/100,000 for Pakistan, and 454.45/100,000 for India.

**Table 2:** Incidence rate (1/100,000) of Salmonella-associated typhoid fever among children aged 5-15 years in China, India, Indonesia, Pakistan, and Viet Nam

	Study sites				
*Salmonella*-associatedtyphoid fever	Hechi, China	Kolkata, India	N. Jakarta, Indonesia	Karachi, Pakistan	Hue, Viet Nam

Laboratory-confirmed incidence of typhoid fever (1/100,000)					
Surveillance period	08/2001∼07/2002	09/2002∼ 10/2004	08/2002∼ 07/2003	08/2002∼ 07/2003∗	07/2002∼ 06/2003
Population	17,124	12,771	32,164	31,727	66,165
Febrile cases	383	1,206	989	5,090	3,405
Typhoid cases	5	58	58	129	16
Mean age (years) of typhoid fever cases	12.04	9.33	10.21	8.61	10.49
Incidence (1/100,000)	29.20	454.15	180.33	407.18	24.18
Perceived prevalence of enteric fever (%)					
Sample size (18-60 years old)	624	561	591	481	1,043
Enteric fever is common	3.8	66.0	26.3	52.7	4.7
Household members suffered from enteric fever	14.0	36.2	52.4	30.5	2.3
Acquaintances suffered from enteric fever	41.6	41.2	48.4	44.0	20.3
Knowing someone died from enteric fever	9.0	6.9	28.3	4.7	5.2

∗Data for one surveillance area were collected during August 2003–July 2004

The perceived prevalence of enteric fever is also presented in Table 2 (lower panel). The percentage of people who perceived enteric fever varied when different questions were used. For example, for the sample from China, only 3.8% reported that enteric fever was common in the wider community, but 14.0% reported having household members who suffered from enteric fever, 41.6% reported having known someone other than household members who suffered from the disease, and 9.0% reported having known someone who died of the disease. In addition, all four measures of perceived prevalence varied dramatically across the study sites. For example, only 2.3% of Vietnamese reported having household members who suffered from enteric fever, whereas 52.5% of Indonesians reported so.

**Fig. 2:**
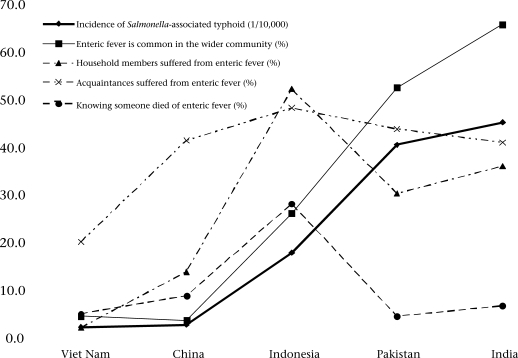
Perceived prevalence of enteric fever by adults appears to be associated with laboratory-confirmed incidence of *Salmonella*-associated typhoid in children

Data in Figure 2 further indicated that, in general, more people from sites with higher rates of typhoid incidence compared to those with lower rates reported that enteric fever was common in the wider community; household members suffered from enteric fever; relatives, friends, and acquaintances suffered from enteric fever; and knowing someone died of enteric fever. For example, people from the study site in Viet Nam where the medically-recorded incidence of typhoid was the lowest, only 4.7% reported that enteric fever was common in the wider community, and 2.3% reported that household members suffered from enteric fever. Among people from the study sites in India where the medically-recorded typhoid fever was the highest, 66.0% reported that enteric fever was common, and 36.2% reported that household members suffered from enteric fever. However, the percentage substantially declined among subjects from Pakistan and India who reported knowing someone who died from enteric fever and a household member suffered from enteric fever, although the observed incidence of typhoid was the highest in these two sites.

### Modelling results

Although data in Table 2 and Figure 2 indicated that the four variables assessing the perceived prevalence of enteric fever appeared to be associated with laboratory-confirmed incidence across the five study sites, results of regression analysis indicated that, for only the perceived prevalence in the wider community (“How often do you think the enteric fever is common in your village/town?”) was this association statistically significant (p<0.05). We, thus, used this variable as X_1_ in the modelling analysis. In addition to the perceived prevalence, per-capita GNI and educational attainment of the population were included to improve the goodness-of-fit of the model to the data. The variable per-capita GNI provided a measure of economic development, which might have contributed to differences in the incidence of typhoid; and the variable education level provided information to account for potential differences in people's perception of enteric fever. Results in Table 3 indicate that, of the six models tested, two linear regression models (models 1(a) and 1(b), p<0.01 for both) and one log linear regression model (model 2(a), p=0.01) fitted the data statistically well according to F-test. Although the statistic R^2^ of the model 2(c) was comparable with that of the model 1(a), the F-test indicated that the model 2(c) did not fit the data statistically well (p>0.05). The model 1(a) was, thus, the most applicable among all the six models because it was statistically significant and is, therefore, reliable. It contained only one predictor variable, and this is practical. The correlation between the levels of perceived prevalence and the rates of laboratory-validated incidence was high (*R*^2^>0.99). Based on the model 1(a), we prepared a prediction equation for practical use:

**Table 3:** Results of different alternative predictive models

Model	Intercept	X_1_	X_2_	X_3_	R^2^	F	p value

Y=incidence p							
Model 1(a)	-2.6946	7.2296∗∗			0.992	358.5	<0.01
Model 1(b)	-6.1183	7.2493∗∗	0.4099		0.992	119.8	<0.01
Model 1(c)	15.9163	7.3812∗	2.6447	-0.6572	0.93	54.5	>0.05
Y=logit (p)							
Model 2(a)	-8.2121∗∗	0.0482∗			0.913	31.7	=0.01
Model 2(b)	-8.7359∗	0.0512∗	0.0625		0.923	12.1	>0.05
Model 2(c)	-7.8482∗	0.0565∗	0.1526	-0.0265	0.997	128.6	>0.05

Y=Medically-recorded incidence of typhoid fever (1/100,1000) among children aged 5-15 years during one year period; X_1_=Perceived prevalence of enteric fever in the wider community or the percentage of subjects who gave an answer of ‘Common’ or ‘Very common’ to the question “How common do you think enteric fever is in your village/town?”; X_2_=Gross national income (in US$ 100); X_3_=Percentage of population with education attainment of less than high school

∗∗p<0.01;

∗p<0.05


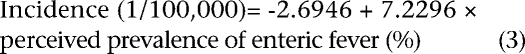


## DISCUSSION

In this study, we have established a simple and cost-effective method to predict the incidence of typhoid using reported data from adults on perceived prevalence of enteric fever in the wider community. According to this model, a 10% increase in perceived prevalence of adults is associated with 0.72296/100,000 increases in incidence of typhoid fever in children. Disease-incidence data in young children are often costly to acquire (as presented in this study), while the obtaining perceived diseaseprevalence data from adults is less costly. The perceived prevalence data can be obtained by simply asking the question, “How common do you think enteric fever is in your village/town?” among randomly-sampled adults. After the percentage of subjects who give a positive answer to this question (either common or very common) is computed, a multiplication of this percentage with the coefficient 7.2296, then minus 2.6946, would provide an estimated incidence rate of typhoid fever. The prediction model is based on data from multisites/nations covering various situations common for many developing countries worldwide, which makes it valid and appropriate for use in countries/regions with similar situations. One limitation of the prediction model is that it is based on a linear equation. Caution is needed when the model is used for predicting the burdens of disease with perceived prevalence beyond the range of 3.8-66%, because this linear regression-based model is more sensitive than the logit linear model on the extreme values of a predictor variable (11,24).

Also important is the finding regarding the relationship between the incidence of disease in children and perceived prevalence in the wider community by adults. With the laboratory-validated incidence of disease, the statistics (*R*^2^>0.99 and significant and p<0.01 for the established model) of this study support our hypothesis that there is a direct relationship between adults’ perception of the frequency of a disease in the wider community and the actual incidence of the disease in children. If the hypothesis of this study can be further verified with new data on the same disease in other regions and other diseases in general, it will provide a unique behavioural epidemiological approach that uses people's perception of a disease, a behavioural science method, to predict the incidence of disease in epidemiology. The method is cost-effective for the control and prevention of disease in the world.

There are several limitations to this analysis. First, although the model can predict the incidence of typhoid, it is based on data only from five study sites in five Asian countries. The prediction of incidences in countries/places with different socioeconomic conditions needs to be verified with new data. In addition, the recorded typhoid-incidence data for age-groups other than 5-15 years are not available across the study sites; therefore, the model developed from the study can only be used for predicting the incidence of typhoid for part of the at-risk population. Second, the ‘best’ model 1(a) is a linear equation. Expanding the range of perceived enteric fever beyond the observed range used in this study may result in an illogical result. For example, the predicted incidence would be minus 2.6946/100,000 if the perceived prevalence of enteric fever in a wider community is zero. Third, cases of typhoid for three study sites (China, Indonesia, and Pakistan) were identified from febrile children who visited the participation clinics. Although great efforts, e.g. media campaign and provision of free care, were devoted to encourage participation, it is likely that not all febrile children from these sites were included because of other barriers, e.g. difficulties in transportation. Fourth, although blood culture was used for collecting evidence on infection due to S. Typhi, it may result in underestimation of incidence of the disease. Results of reported studies indicated that it is the bacterium in the bone marrow not the blood that reflect the clinical course of the infection (19,20). Using one-time blood culture might have resulted in underestimation of incidence of the disease. Overestimation was also possible because results of studies among paediatric patients have indicated that isolation of S. Typhi in blood through culture may not necessarily reflect clinical infection (21). Fifth, it is possible that, if one area had a specially-intense programme of obtaining blood cultures among febrile patients and that this increased intensity was in areas with a higher prevalence of typhoid fever, this association could result in an increased awareness of typhoid fever in the high-density areas. However, we have no information regarding the ongoing prevalence of obtaining blood cultures by local hospitals in various study countries and no evidence that this speculation is correct. Lastly, attention should be paid to the two measures of socioeconomic status, e.g. GNI and education attainment, that are intuitively related but did not significantly predict typhoid fever in this study. It is not clear whether this zero effect is due to inaccuracy of measurement of these variables, and/or other confounders. Future studies should include these and other related factors, e.g. lifestyle, sanitary conditions, and overcrowding, in modelling.

Despite these limitations, the model-predicted incidence data provide a cost-effective alternative for planning prevention and control of typhoid fever in developing countries where there is a growing need for such data by policy-makers and vaccine producers but where data on laboratory-validated typhoid incidence are either too costly or not readily available (6,25). Compared to laboratory-based approach, there are several advantages with the method we developed in this analysis. Our method can be used in countries/regions without adequate technical and/or financial resources for conducting laboratory-confirmation studies. Data needed for this prediction consist of only one simple question regarding the perceived prevalence of enteric fever; the question can be easily added to existing surveys, which are commonly used in public-health practice in many developing countries (5,6,15-17). In addition, data for control of disease can be acquired much faster using our method than laboratory-validated surveillance method. More studies with consistent results are needed to convince policy-makers and stakeholders to use this method and data in practice.
